# Supplementary material for: High-order coherent communications using mode-locked dark-pulse Kerr combs from microresonators
Source: Nat Commun. 2018 Apr 23;9:1598. doi: 10.1038/s41467-018-04046-6 (PMC5913129; doi:10.1038/s41467-018-04046-6)
Supplement: Supplementary file 1 — Supplementary Information [file 41467_2018_4046_MOESM1_ESM.pdf]

High-order coherent communications using mode-locked dark-pulse Kerr combs from microresonators: Supplementary Information

Fülöp et al.

Supplementary Note 1: Fabrication feasibility of the optimized comb

In order to evaluate the fabrication feasibility of the waveguide suggested in Fig. 4 in the main paper, we performed cross-section simulations using a finite element method-based mode solver (COMSOL). We calculated the chromatic dispersion for a variety of realistic rectangular waveguide geometries for wavelengths around λ=1550 nm. Supplementary Figure 1 shows that using rectangular geometries, it is indeed possible to achieve the optimal dispersion value for the fundamental waveguide mode using several width and height combinations. The material dispersion for silicon nitride used in the simulations was taken from refs. 1,2. As the comb initialization strongly depends on the interaction between two co-polarized modes[3], the part of the valid contour supporting exactly two X-polarized modes has been highlighted.

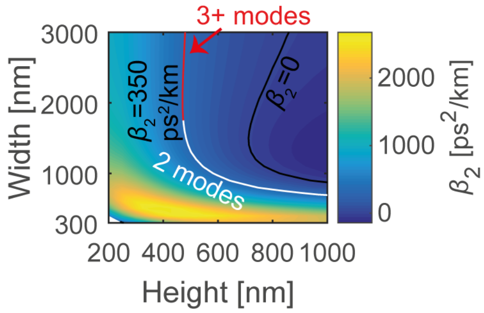

Supplementary Figure 1 | Dispersion of rectangular waveguides. Simulated group-velocity-dispersion coefficient, β_2, for rectangular waveguides with realistic sizes at λ=1550 nm. The contours along the lines show the geometries giving β_2=350 ps^2/km and β_2=0 ps^2/km respectively. The white-marked part of the 350 ps^2/km line indicates the region where the waveguide supports exactly two X-polarized modes.

In the following, we illustrate that the power coupling parameter, θ, obtained in the simulations is also achievable in realistic geometries. Using the same mode solver as above together with standard coupled mode equations it is possible to evaluate the coupling constant κ between the fundamental modes of two adjacent waveguides[4,5]. Supplementary Figure 2a illustrates how this constant is varying for a twin waveguide system (with 1000 nm x 600 nm waveguides) as the gap distance is swept. This geometry provides a solution to optimize the coupling ideality when the microresonator sustains more than one mode[6]. Depending on the achievable minimum gap during fabrication, this will translate into a varying required length of the coupling region as illustrated in Suppl. Fig. 2b. As the resonator circumference for a 100 GHz resonator is more than one millimeter, all the shown coupling lengths are feasible with standard microlithographic tools.

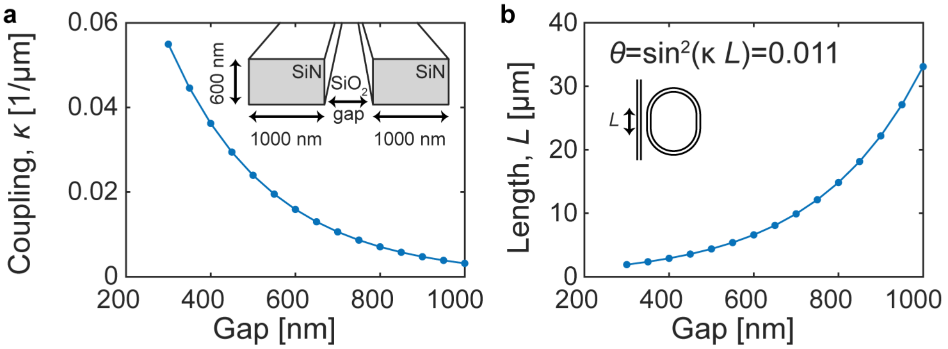

Supplementary Figure 2 | Coupling between bus and ring waveguides. (**a**) Simulated coupling parameter, κ, for identical waveguides with 1000 nm x 600 nm dimensions with varying gap distance. The inset shows a sketch of the simulated twin waveguide configuration. (**b**) Coupling region length needed for a power coupling constant $\theta=\sin^2(\kappa L)=0.011$ as indicated in Fig. 4a in the main paper. The inset shows a top-down view of the suggested geometry.

Supplementary Note 2: Comb bandwidth and line spacing

In the discussion section of the main paper, we found the optimum 100 mW-pumped C-band-spanning 100 GHz spaced dark pulse comb. It is however possible to optimize for other scenarios as well. Dark pulse combs can be designed to operate with different pump powers, narrower spacing as well as covering a wider bandwidth. In Suppl. Fig. 3a we show corresponding optimal comb states for 50 GHz and 100 GHz combs covering the C-band (1530 nm – 1565 nm) and C+L bands (1530 nm – 1610 nm). The optimization process was identical to the one done in the main paper as described in the main Methods section. As predicted for other microresonator comb states[7], increasing the number of generated comb lines will lower the reachable conversion efficiency as well as the power available in the weakest line.

In Suppl. Fig. 3b we have varied the pump power showing that dark pulse comb states remain viable multi-wavelength light sources with pump powers below 20 dBm. A 3 dB decrease in pump power translates to a 4-6 dB decrease in the weakest line power. At the low power end, a 25 mW pump will be enough to give a C-band spanning 100 GHz comb a minimum line power of -20 dBm. This corresponds to 38 dB above the quantum-limited noise floor. In principle, this is enough margin to allow for PM-64QAM modulation, assuming the surrounding equipment does not further decrease the limit. Should further margins be required, one can relax the received OSNR requirement by using more advanced, soft-decision, error correction schemes[8].

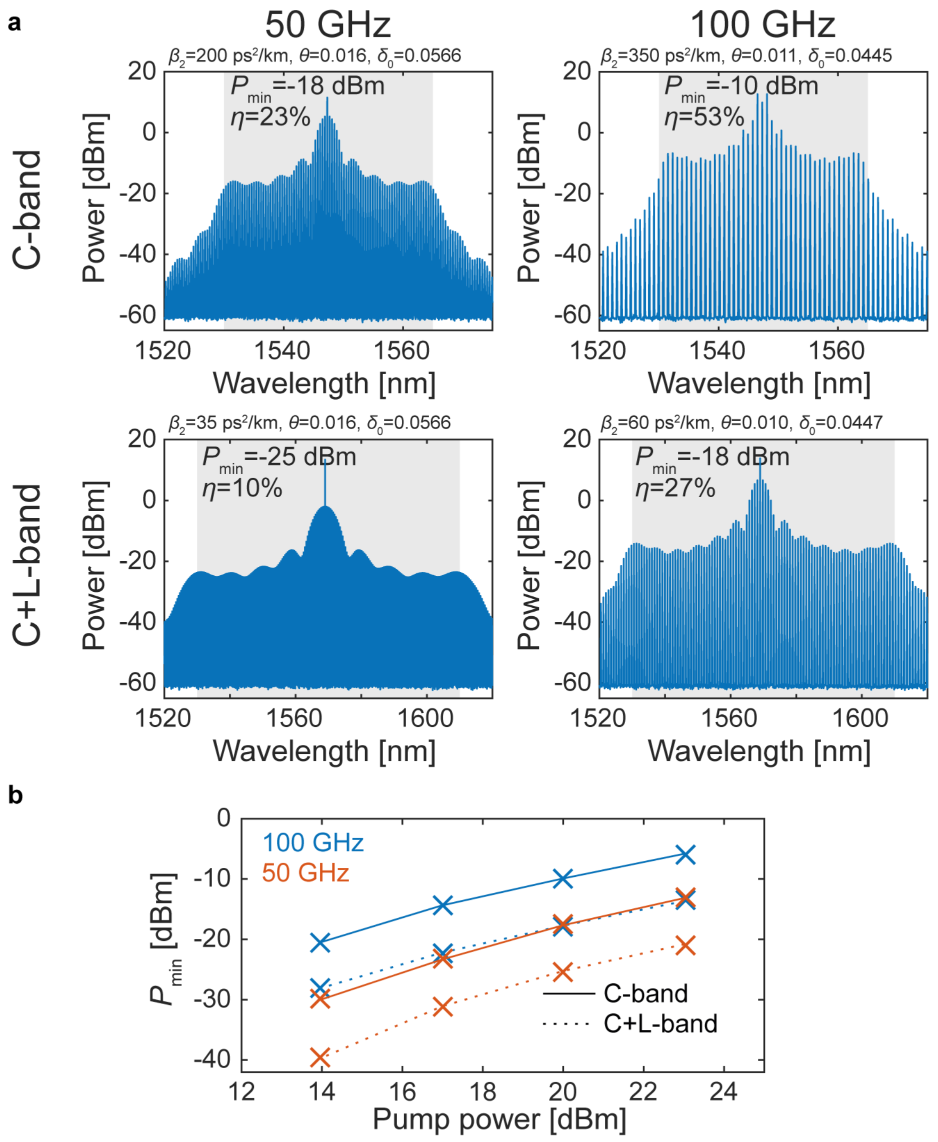

Supplementary Figure 3 | Simulated combs with varying bandwidth and line spacing. (**a**) Optimal dark pulse combs with 50 GHz and 100 GHz line spacing spanning both C-band and C+L-bands. The weakest line powers, P_{min}, as well as the total conversion efficiency, η, is calculated. (**b**) The lowest lines' powers, P_{min}, for optimized combs decrease with increasing comb bandwidth and decreasing comb line spacing.

Supplementary Note 3: Conversion efficiency utilization

In the main paper, to overcome the limitations of using two modulators used for the even and odd channels, the power in all comb lines was equalized and amplified before data modulation. This design does not make efficient use of the extra power in the strongest comb lines however, thereby decreasing the net conversion efficiency from >20% to 1.5%. In a scenario where one has access to a separate modulator for each comb line, alternative designs exist that can make better use of the available power. Supplementary Figure 4a displays an example system where the available power is instead translated to higher optical signal-to-noise ratio in the central channels. In those channels, the high OSNR would allow for data to be encoded with higher order modulation formats or lower coding overhead. Note that the

channel powers will still be equal as they are flattened after the first transmitter EDFA. The design does not include active components (such as amplifiers) between the comb source and the modulators, thus simplifying future integration. The sketched system connects the optimum simulated comb from the main paper (Fig. 4b) with transmitter components. By assuming combined multiplexing, demultiplexing and modulator losses of 16 dB and an EDFA noise figure of 5 dB, the dual-polarized data-carrying channels will have OSNRs between 27 dB and 50 dB, see Suppl. Fig. 4b. In this scenario, the weakest lines have slightly lower OSNR than the ones demonstrated in the main paper. The example system however simultaneously allows efficient usage of the comb's conversion efficiency while requiring no active components until the final fiber connection.

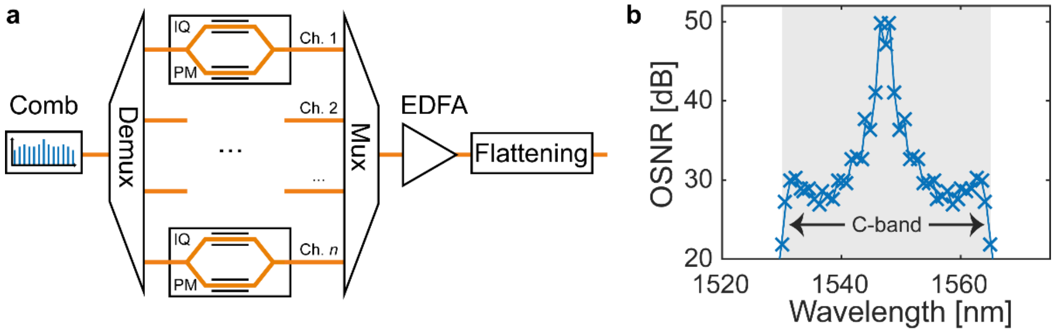

Supplementary Figure 4 | Comb-based transmitter sketches. (**a**) Setup with flattening stage after modulation allowing the line power differences to be translated to OSNR differences in the transmitted channels. (**b**) Resulting OSNR (at 0.1 nm resolution) variation for the simulated comb in the main paper assuming 16 dB losses in the transmitter stage and a 5 dB noise figure EDFA.

Supplementary References

1. Philipp, H. R. Optical Properties of Silicon Nitride. *J. Electrochem. Soc.* **120,** 295 (1973).

2. Bååk, T. Silicon oxynitride; a material for GRIN optics. *Appl. Opt.* **21,** 1069–1072 (1982).

3. Liu, Y. *et al.* Investigation of mode coupling in normal-dispersion silicon nitride microresonators for Kerr frequency comb generation. *Optica* **1,** 137–144 (2014).

4. Haus, H. A., Huang, W.-P., Kawakami, S. & Whitaker, N. A. Coupled-mode theory of optical waveguides. *J. Light. Technol.* **5,** 16–23 (1987).

5. Chin, M. K. & Ho, S. T. Design and modeling of waveguide-coupled single-mode microring resonators. *J. Light. Technol.* **16,** 1433–1446 (1998).

6. Pfeiffer, M. H. P., Liu, J., Geiselmann, M. & Kippenberg, T. J. Coupling Ideality of Integrated Planar High-Q Microresonators. *Phys. Rev. Appl.* **7,** 24026 (2017).

7. Bao, C. *et al.* Nonlinear conversion efficiency in Kerr frequency comb generation. *Opt. Lett.* **39,** 6126–6129 (2014).

8. Alvarado, A., Agrell, E., Lavery, D., Maher, R. & Bayvel, P. Replacing the Soft-Decision FEC Limit Paradigm in the Design of Optical Communication Systems. *J. Light. Technol.* **33,** 4338–4352 (2015).
